# Promoter hypermethylation-mediated inactivation of multiple Slit-Robo pathway genes in cervical cancer progression

**DOI:** 10.1186/1476-4598-5-16

**Published:** 2006-05-15

**Authors:** Gopeshwar Narayan, Chandra Goparaju, Hugo Arias-Pulido, Andreas M Kaufmann, Achim Schneider, Matthias Dürst, Mahesh Mansukhani, Bhavana Pothuri, Vundavalli V Murty

**Affiliations:** 1 Department of Pathology, College of Physicians & Surgeons of Columbia University, New York, NY, USA; 2 Department of Tumor Molecular Biology. Instituto Nacional de Cancerología, Bogota, Colombia and Departments of Molecular Genetics and Microbiology, University of New Mexico, Albuquerque, New Mexico, USA; 3 Charité Universitätsmedizin Berlin, Campus Benjamin Franklin, Klinik für Gynäkologie mit Hochschulambulanz, Hindenburgdamm 30, 12200 Berlin, Germany; 4 Department of Obstetrics & Gynecology, Friedrich Schiller University, Jena, Germany; 5 Gynecologic Oncology, College of Physicians & Surgeons of Columbia University, New York, NY, USA; 6 Institute for Cancer Genetics, College of Physicians & Surgeons of Columbia University, New York, NY 10032, USA

## Abstract

**Background:**

Cervical Cancer (CC) exhibits highly complex genomic alterations. These include hemizygous deletions at 4p15.3, 10q24, 5q35, 3p12.3, and 11q24, the chromosomal sites of Slit-Robo pathway genes. However, no candidate tumor suppressor genes at these regions have been identified so far. Slit family of secreted proteins modulates chemokine-induced cell migration of distinct somatic cell types. Slit genes mediate their effect by binding to its receptor Roundabout (Robo). These genes have shown to be inactivated by promoter hypermethylation in a number of human cancers.

**Results:**

To test whether Slit-Robo pathway genes are targets of inactivation at these sites of deletion, we examined promoter hypermethylation of *SLIT1*, *SLIT2*, *SLIT3*, *ROBO1*, and *ROBO3 *genes in invasive CC and its precursor lesions. We identified a high frequency of promoter hypermethylation in all the Slit-Robo genes resulting in down regulated gene expression in invasive CC, but the inhibitors of DNA methylation and histone deacetylases (HDACs) in CC cell lines failed to effectively reactivate the down-regulated expression. These results suggest a complex mechanism of inactivation in the Slit-Robo pathway in CC. By analysis of cervical precancerous lesions, we further show that promoter hypermethylation of Slit-Robo pathway occurs early in tumor progression.

**Conclusion:**

Taken together, these findings suggest that epigenetic alterations of Slit-Robo pathway genes (i) play a role in CC development, (ii) further delineation of molecular basis of promoter methylation-mediated gene regulation provides a potential basis for epigenetic-based therapy in advanced stage CC, and (iii) form epigenetic signatures to identify precancerous lesions at risk to progression.

## Background

Metastasis and treatment failure is a significant cause of death in invasive Cervical Cancer (CC). Although combination chemotherapy with cisplatin as a primary agent has been commonly used in CC, the overall survival rate did not significantly improve [1]. Despite the obvious role of invasion and metastasis in treatment failure of CC, the molecular mechanisms remain poorly understood. A wide number of genes implicated in metastasis that play role in the migration of tumor cells have been identified [2]. In particular, chemokines that contribute to tumor cell invasion and growth plays a major role in metastasis [3]. Recently, a regulatory molecular pathway involving proteins of Slit-Robo genes has been shown to modulate chemokine-induced leukocyte migration [4,5]. The Slit family of secreted proteins has been identified as molecular guidance cues including cell migration. Slit genes mediate their effect by binding to its receptor Roundabout (Robo) and by an intracellular signal transduction pathway that includes the Abelson kinase, the Enabled protein, GTPase activating proteins, and the Rho family of small GTPases [6]. Interestingly, Slit also appears to use Roundabout to control leukocyte chemotaxis besides neuronal migration, suggesting a fundamental conservation of mechanisms guiding the migration of distinct types of somatic cells [6].

Recent studies show that Slit-Robo pathway genes are inactivated by promoter hypermethylation in a number of tumor types [7-11]. The chromosomal regions that map Slit-Robo pathway genes have been shown to be frequently deleted in CC [12,13]. We hypothesize that the Slit-Robo pathway genes may be targets of inactivation by a combination of deletion and epigenetic mechanisms in CC. In order to test this, we have investigated five genes in this pathway for epigenetic changes during CC progression.

## Results and discussion

The chromosomal bands 4p15.3 (*SLIT2*), 10q24 (*SLIT1*), 5q35 (*SLIT3*), 3p12.3 (*ROBO*1 and *ROBO2*), and 11q24.2 (*ROBO3 *and *ROBO4*) that Slit-Robo pathway genes are located have been previously shown to be frequent targets of LOH in CC [12,13]. To identify if the Slit-Robo pathway genes are targets of chromosomal deletions, we chose to examine loss of heterozygosity (LOH) in the vicinity of *SLIT2 *at 4p15.3 and *ROBO1*/*ROBO2 *at 3p12.3 regions, the two most critical genes in the pathway. We performed LOH in 30 primary tumors using STS markers (D4S1593, D4S1562, D4S2946, D4S1525, D3S1542, D3S3681, D3S3031, and D3S3508) mapped close to these genes. This analysis found hemizygous deletions of one or more of these loci in only 9% and 10% of CC at 4p15.3 and 3p12.3, respectively (data not shown). This data, thus, suggests that genomic regions spanning *SLIT2 *and *ROBO1/ROBO2 *genes are not frequent targets of LOH in CC. Because of the recent reports of promoter hypermethylation of *SLIT2 *and *ROBO1 *genes in multiple tumor types [7-9,11,14], we reasoned that this family of genes may be targets of epigenetic inactivation in CC. To test this hypothesis, we examined the status of hypermethylation of *SLIT1*, *SLIT2*, *SLIT3*, *ROBO1*, and *ROBO3 *genes that harbor CpG islands in their promoters in CC progression.

### Slit-Robo pathway genes are concomitantly hypermethylated in invasive CC

**Figure 1:**
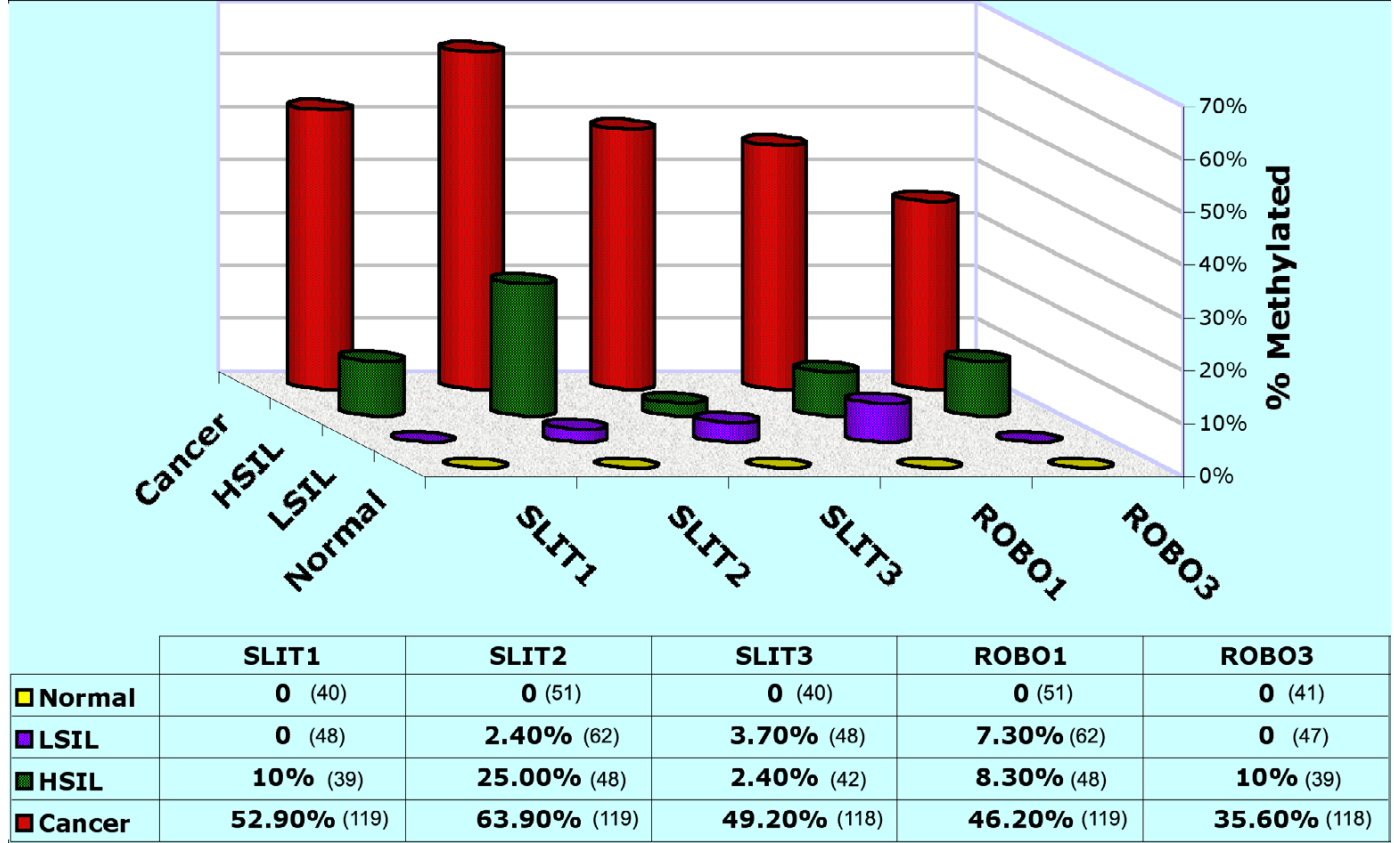
Frequency of promoter hypermethylation of Slit-Robo pathway genes in cervical cancer progression. High molecular weight DNA isolated from pap smears and tissue sections was converted by sodium bisulphite [15]. MS-PCR was performed on converted DNA using primers specific to methylated and umethylated templates of each gene (Table 1). PCR products were separated on 2% agarose gels and visualized after ethidium bromide staining. Promoter methylation was scored on gels in the presence of positive and negative controls in each experiment. LSIL, low-grade squamous intraepithelial lesion; HSIL, high-grade squamous intraepithelial lesion. The total number of specimens analyzed in each type of tissue and gene are shown in parenthesis in the table below.

**Figure 2:**
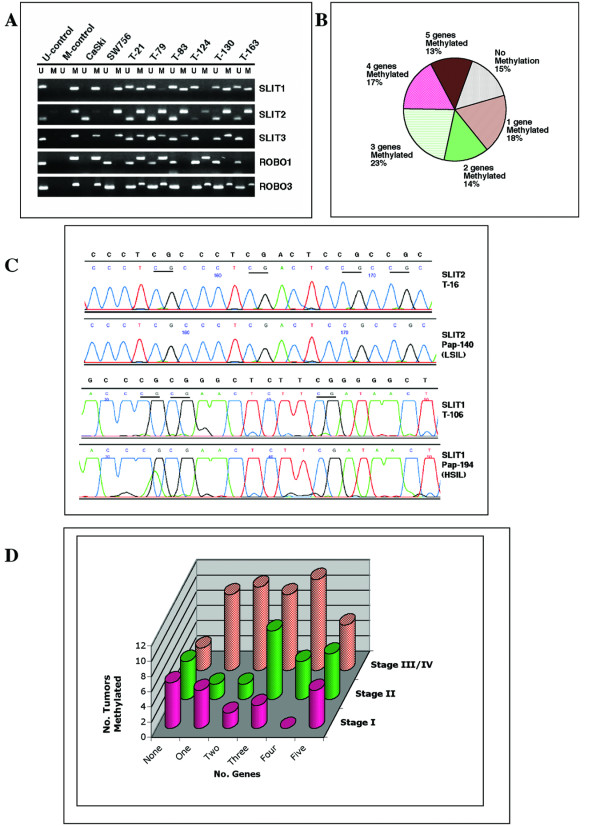
Analysis of methylation of Slit-Robo pathway genes in cervical cancer cell lines and primary tumors. **A**. MSP analysis. U, unmethylated; M, methylated. T, tumor. **B**. Concomitant hypermethylation of more than one Slit-Robo genes in primary cervical cancer. Frequency of number of genes methylated is shown. **C**. Sequence analysis of MSP products of *SLIT1 *and *SLIT2 *genes. *SLIT2 *sequences were derived from cloning of PCR products and *SLIT1 *was direct sequencing of MSP products. T, tumor; pap, cytologic smear. CpG sites are underlined. Unconverted sequence is shown above chromatogram for each gene. **D**. Number of Slit-Robo genes methylated in various stages of invasive cervical cancer.

To evaluate the methylation status of *SLIT1*, *SLIT2*, *SLIT3*, *ROBO1*, and *ROBO3 *gene promoters, we employed the methylation-specific PCR (MSP) method that qualitatively assess the presence or absence of hypermethylation of a small number of CpG sites within the promoter [15]. Primers used for this analysis are shown in Table 1. Such an analysis on 51 specimens obtained from normal cervical epithelia did not show any evidence of promoter hypermethylation in *SLIT1*, *SLIT2*, *SLIT3*, *ROBO1*, and *ROBO3 *genes. These data, thus, suggest that Slit-Robo pathway genes are in unmethylated state in normal squamous epithelium of cervix. However, our analysis of 119 DNAs derived from CC (9 cell lines and 110 primary tumors) identified a high frequency of promoter hypermethylation of these genes ranging between 35.6–63.9% tumors (Figures 1 and 2). *SLIT2 *was the most frequently (76 of 119 tumors; 63.9%) methylated gene. Promoter hypermethylation of *SLIT1 *in 52.9% (63 of 119 tumors), *SLIT3 *in 49.2% (58 of 118 tumors), *ROBO1 *in 46.2% (55 of 119 tumors), and *ROBO3 *in 35.6% (42 of 118 tumors) cases was found.

**Table 1:** Primers used for MSP, RT-PCR, and cloning and sequencing.

**MSP primers**:		
SLIT1-MF2	5'-TtcgTtcgcgagTTagacg-3'	19 bp
SLIT1-MR2	5'-aAAcgccgtcgcttAAaAA-3'	19 bp
SLIT1-UF2	5'-TgggTttgTgTgTggTgTTT-3'	20 bp
SLIT1-UR2	5'-ttttcctcctcAcaAcaAtcaA-3'	22 bp
SLIT2-MF	5'-gggaggcgggattgTTTag-3'	19 bp
SLIT2-MR	5'-catAAcgcgcgAAAAtAcac-3'	20 bp
SLIT2-UF	5'-gTgggaggTgggattgTTTa-3'	20 bp
SLIT2-UR	5'-AcctctccctcAccctcAac-3'	20 bp
SLIT3-MF	5'-ggtttcgtcgatggagttgt-3'	20 bp
SLIT3-MR	5'-aaacgcgtaaaacccgaaa-3'	19 bp
SLIT3-UF	5'-TGTGggTTagTGgggTTagg-3'	20 bp
SLIT3-UR	5'-cacaaacaaaacaaaacactcca-3'	23 bp
ROBO1-MF2	5'-cggcggcgatagTagTTaaa-3'	20 bp
ROBO1-MR2	5'-cgAAActAAAAAcgcccaAa-3'	20 bp
ROBO1-MF3	5'-cggcgtgcgTTTTTaTaatg-3'	20 bp
ROBO1-MR3	5'-gccAcgAAtAAcccgctAct-3'	20 bp
ROBO1-UF	5'-TggTggTaaagttggggtgt-3'	20 bp
ROBO1-UR	5'-ccAaAcccttcctccAAaAc-3'	20 bp
ROBO3-MF	5'-gcgggaTtTtTagTcggTTT-3'	20 bp
ROBO3-MR	5'-gAcctctccgcaAActAAcg-3'	20 bp
ROBO3-UF	5'-TggTgggaTtTtTagTTggTTT-3'	22 bp
ROBO3-UR	5'-ccAcaActtccccAcAAcAc-3'	20 bp
**RT-PCR primers**:		
SLIT1-F	5'-ctggaactcaatggcaacaa-3'	20 bp
SLIT1-R	5'-acaaagcctggttgttctgg-3'	20 bp
SLIT2-F	5'-tcagctgtttcctgagttgc-3'	20 bp
SLIT2-R	5'-tggttgaaacttgccacaga-3'	20 bp
SLIT3-F	5'-gcgcctgaacaagaataagc-3'	20 bp
SLIT3-R	5'-ggatgcgactgatgttgttg-3'	20 bp
ROBO1-F1	5'-tgtttctggcccagcttatt-3'	20 bp
ROBO1-R1	5'-gtgttcaacaatgcgaggtg-3'	20 bp
ROBO1-F2	5'-aaatatggtgggcaaagctg-3'	20 bp
ROBO1-R2	5'-ctggatgactgtggtggttg-3'	20 bp
ROBO3-F	5'-gcagtcctccgtgatgattt-3'	20 bp
ROBO3-R	5'-ttggaggctacgcacacata-3'	20 bp
**Cloning Primers**:		
SLIT2-cl-F	5'-gaattTaaagTTtgggaaaagttg-3'	24 bp
SLIT2-cl-R	5'-cttccaacaactactaaaatacaaaaa-3'	27 bp
SLIT2-cl-F3	5'-agtgTtgaTtagtggatatttTtgTT-3'	26 bp
SLIT2-cl-R3	5'-tcttctAtctcccaaAAatAaactt-3'	25 bp

Nucleotides shown in upper case represent converted bases; MF and MR, methylation-specific forward and reverse primers; UF and UR, unmethylated forward and reverse primers; F and R, forward and reverse primers; cl-F and cl-R, cloning forward and reverse primers

Promoter hypermethylation of *SLIT2 *ranging in frequency between 25–72% has been reported in a broad spectrum of tumors such as colon, glioma, lung, breast, renal cell cancer, Wilms tumor, and neuroblastoma [8,9,11,16]. Promoter hypermethylation of other Slit-Robo pathway genes has not been extensively studied in cancer. *SLIT3 *gene promoter hypermethylation ranging from 7–41% has been shown in tumors arising from carcinomas of lung, breast, colon, and glioma [16]. Promoter hypermethylation of *SLIT1 *gene reported to be present in 10% of gliomas [16]. The *ROBO1 *gene promoter methylation has been found in 4–19% in lung, breast, and renal cell carcinomas [7]. *ROBO3 *gene promoter methylation has not been reported in cancer so far. In the present study, we identified promoter hypermethylation in all five Slit-Robo pathway genes examined and the observed frequency of methylation is the highest in any tumor type reported thus far. One or more genes in this pathway exhibited promoter hypermethylation in 85% of CC cases suggesting a major role for the Slit-Robo pathway in this cancer. Three or more genes showed promoter hypermethylation in 53% of the tumors studied. Among the 101 tumors with promoter hypermethylation, 16 (13%) showed methylation of all five genes (Fig. 2A and 2B). To further confirm MSP results and to assess the extent of methylation of CpG sites, we performed sequence analysis on representative tumors either by direct sequencing of PCR products or sequencing followed by cloning PCR products. We found consistent results by both methods in all tested cases (Fig. 2C). Furthermore, the sequencing data provided a qualitative estimate of methylation of CpG sites in all five genes examined. The extent of CpG methylation varied among the genes tested in invasive cancer and precancerous lesions. *SLIT1 *gene showed 87.5–93.8% methylated CpG sites, *SLIT2 *exhibited 100% CpG site methylation, *SLIT3 *showed 40.7–100%, *ROBO1* showed 41.7–100%, and *ROBO3 *showed 87.5% CpG site methylation. We did not notice any substantial differences in the number of CpG sites methylated between invasive cancer and precancerous lesions. Thus, this data provide evidence for Slit-Robo pathway genes as targets of promoter hypermethylation in CC and the concomitant methylation of multiple genes further suggest a complex mechanism of inactivation of this pathway in CC tumorigenesis.

In order to further examine the role of Slit-Robo genes in CC, we performed a correlative analysis of hypermethylation with clinico-pathologic features such as age, tumor stage and size of the tumor, clinical outcome, and HPV type in primary tumors. No significant differences were found when individual genes were examined (data not shown). No significant differences in promoter hypermethylation between cell lines and primary tumors were found (data not shown). However, we found that advance stage tumors (stages III and IV) exhibit a significantly (p < 0.025) higher frequency of promoter methylation in 2 or more Slit-Robo family genes compared to early stage (stages I and II) tumors (Fig. 2D). These data therefore suggest that concomitant promoter hypermethylation and inactivation of multiple Slit-Robo pathway genes play a role in progression of CC.

The presence of concordant high frequency of promoter hypermethylation of Slit-Robo pathway genes in CC is reminiscent of the CpG Island Methylator Phenotype (CIMP) in cancer [17]. The CIMP phenotype can be caused by exposure to epimutagens, which potentially target gene-specific methylation in a cancer-specific manner [18,19]. Infection of high-risk human papillomavirus (HPV) is known to be primary cause of CC [20]. In the present study, we did not find any significant correlation between methylation frequency and various HPV types in CC. However, a controlled study comprising a large number of HPV-negative tumors is required to completely rule out the role for HPV in Slit-Robo pathway gene methylation. Although the causes of CIMP remain poorly understood, a significant correlation of DNA methyltransferases (DNMTs) expression with DNA hypermethylation of multiple CpG islands has been shown [21]. DNMTs have been also shown to be generally over express in cancer and play a role in aberrant DNA methylation [17,22]. To examine the role of DNMT expression in Slit-Robo pathway gene methylation, we examined the expression levels of *DNMT1*, *DNMT3a*, and *DNMT3b *by semi-quantitative RT-PCR analysis. We identified over expression of all three tested DNMTs in CC. *DNMT1 *over expression was found in all CC cases (100%), whereas the *DNMT3a *(73.7%) and *DNMT3b *(78.9%) genes were over expressed to a lesser extent in CC cases (Fig. 4). However, this over expression of DNMTs showed no significant correlation with promoter hypermethylation of Slit-Robo pathway genes (data not shown), and therefore no relationship between these molecular alterations could be established.

**Figure 3:**
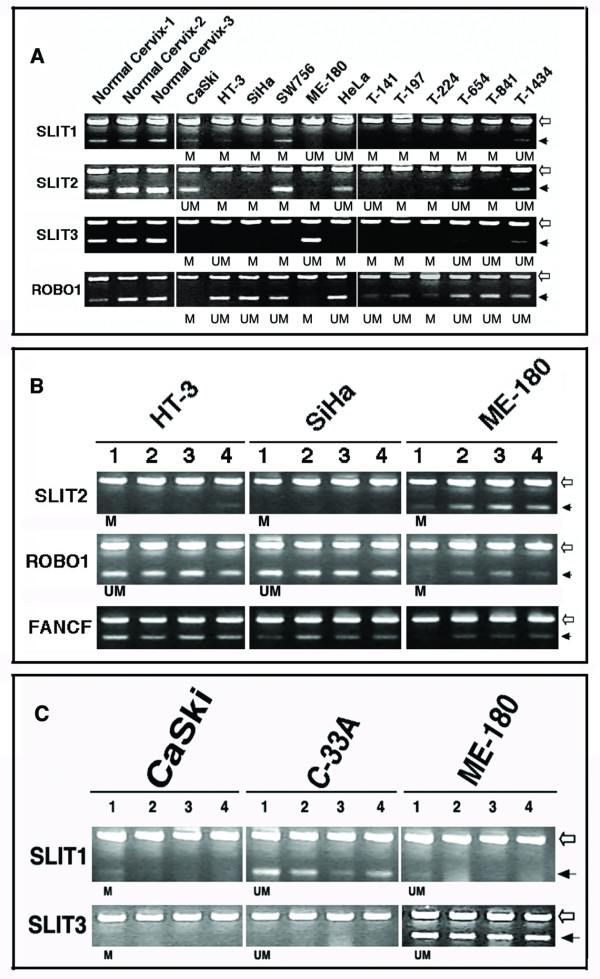
Analysis of Slit-Robo pathway gene expression by RT-PCR in cervical cancer cell lines and primary tumors. **A**. Expression of *SLIT1*, *SLIT2*, *SLIT3*, and *ROBO1 *genes. Note the high-levels of expression in normal cervix, complete loss or down-regulated expression in the cell lines (CaSki, HT-3, SiHa, SW756, ME-180, and HeLa) and primary tumors (shown by prefix "T" for tumor). **B-C**. Effect of demethylation and acetylation on *SLIT2*, *ROBO1*, *SLIT1*, and *SLIT3 *genes. *FANCF *gene is shown as a control for reactivation of expression in SiHa and ME-180 in panel B [31]. Lanes 1, untreated; 2, 5-aza-CdR-treated (5 or 10 μM for 5 days); 3, TSA treated (100 nM for the last 24 hours); 4, 5-aza-CdR and TSA treated. Note that *SLIT2 *promoters were methylated in all three-cell lines but only ME-180 showed reactivation, while HT-3 showed minimal reactivation only in combined 5-aza-CdR and TSA treated cells but not with other treatments. SiHa failed to reactivate. For *ROBO1 *gene, ME-180 had methylated promoter and showed reactivated expression with all treatments. No reactivation of *SLIT1 *and *SLIT3 *genes in promoter methylated CaSki cell line was found. Beta actin (empty arrow) used as an internal control; Filled arrows indicate specific genes used for RT-PCR. Promoter methylation status of each gene is shown below the panels.

**Figure 4:**
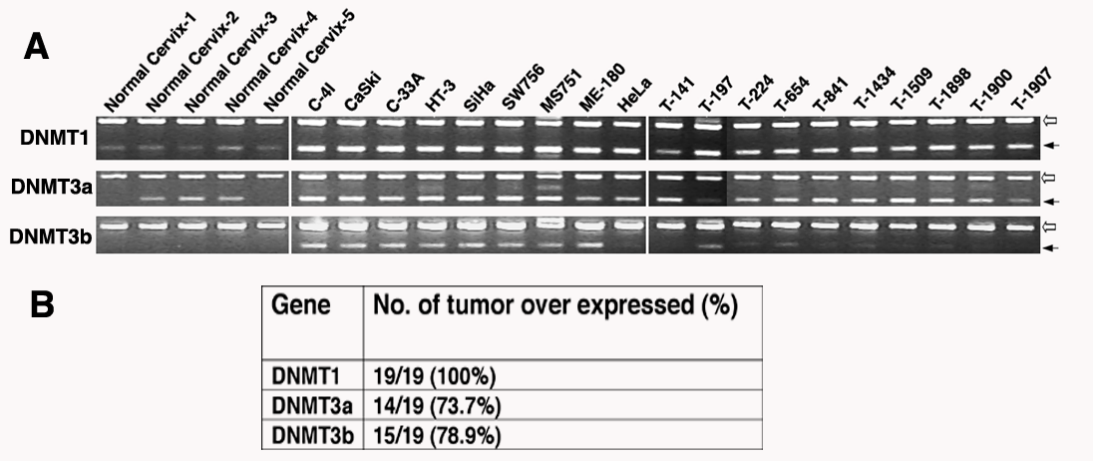
Analysis of expression of DNMT genes in normal cervix, cervical cancer cell lines, and primary tumors. **A**. Multiplex RT-PCR analysis of *DNMT1*, *DNMT3a*, and *DNMT3b *genes. Prefix "T" indicates primary tumor. Beta actin (empty arrow) used as an internal control; Filled arrows indicate specific genes used for RT-PCR. **B**. Table showing the frequency of over expression of each of the genes based on semi-quantitative analysis.

Slit2 inhibits chemotaxis and chemoinvasion by down-modulating down-stream signaling molecules CXCR4/CXCL12 and CXCL12-induced phosphatidylinositol 3 kinase [23] and Slit-2 protein can inhibit the migration of endothelial cells lacking Slit-2 [5]. Therefore, the epigenetic silencing of multiple Slit-Robo pathway genes may play a role in invasive potential of CC cells. Based on the functions of Slit-Robo family genes and our observations raise a number of questions: i) what is the role of inactivation of both receptor and ligand in CC tumorigenesis? ii) Is there an upstream regulator of promoter methylation of Slit-Robo pathway genes in CC? iii) Are there any down-stream effectors of Slit-Robo methylation that affect invasion and migration of CC cells?

### Promoter hypermethylation of Slit-Robo pathway genes is an early event in tumor progression

To identify the role of promoter hypermethylation of Slit-Robo genes in CC progression, we studied DNA obtained from 110 cytological smears diagnosed as low-grade squamous intraepithelial lesions (LSIL) in 62 and high-grade SIL (HSIL) in 48 cases by MSP. We found evidence of promoter hypermethylation in at least one gene in 11 of 62 (17.7%) LSIL and 15 of 48 (31.3%) HSIL, which suggests that Silt-Robo pathway genes are methylated early in CC progression. Among the LSILs, a low frequency of hypermethylation occurs in *SLIT2*, *SLIT3*, *ROBO1*, whereas *SLIT1 *and *ROBO3 *showed no methylation. While the promoter hypermethylation of *SLIT1*, *SLIT3*, *ROBO1*, and *ROBO3 *genes were low in HSIL, the *SLIT2 *gene showed higher frequency of hypermethylation in 12 of 48 (25%) cases (Fig. 1). This data suggest that *SLIT2 *inactivation is an early and a primary event, while the methylation of the other genes in the pathway occur later in the progression. The natural history of cervical precancerous lesions varies with approximately 1% of low-grade and 15% of high-grade Cervical Intraepithelial Neoplastic lesions progress to invasive cancer [24], and therefore, the epigenetic changes documented here may form potential signatures to identify precancerous lesions at high-risk to progress to invasive cancer. However, analysis of a larger cohort of precancerous and cancerous lesions is needed to validate such a hypothesis.

### Down regulated expression of Slit-Robo pathway genes in relation to promoter hypermethylation and inefficient reactivation after exposure to inhibitors of methylation and histone deacetylases

Although the Slit-Robo family proteins primarily express in the developing nervous system, they also widely express outside the nervous system in adult tissues suggesting roles outside the developing embryo [25]. Consistent to this, we found that all three Slit genes and *ROBO1 *are ubiquitously expressed in normal cervical tissues (Fig. 3A). However, no detectable expression of *ROBO3 *in normal cervix or in CC cell lines by RT-PCR was found and thus this gene was not studied for expression. To further test the role of promoter hypermethyation of *SLIT1*, *SLIT2*, *SLIT3*, and *ROBO1 *genes in CC, we studied the expression by semi-quantitative RT-PCR analyses in nine CC cell lines and 10 primary tumors. A complete loss of or down regulated expression was found in the majority of cases with promoter hypermethylation of *SLIT2 *(9 of 11; 81.8%), *SLIT1 *(8 of 11; 72.7%), *SLIT3 *(11 of 11; 100%), and *ROBO1 *(6 of 7; 87.5%) genes compared to normal cervices (Fig. 3A). Overall, the down-regulated expression correlate with promoter hypermethylation and these results suggest that epigenetic promoter methylation play a role in inactivating Slit-Robo pathway genes in CC.

**Figure 5:**
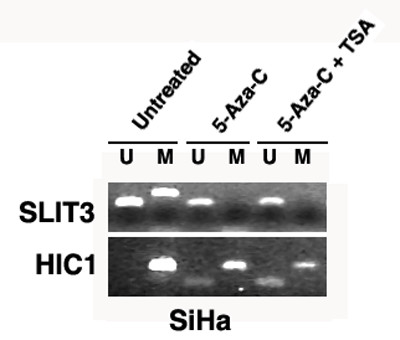
Effect of inhibition of DNA methylation by 5-aza-CdR and TSA-treatment on *SLIT3 *promoter in SiHa cell line. Biallelically methylated *HIC1 *gene was used as control [29]. U, unmethylated primer; M, methylated primer; Note the absence of methylated allele after treatment with 5-aza-CdR, and 5-aza-CdR+TSA of *SLIT3 *gene (top panel). Note the decreased intensity of methylated allele and reappearance of unmethylated allele of *HIC1 *after 5-aza-CdR, and 5-aza-CdR+TSA treatments (bottom panel).

DNA hypermethylation-mediated gene silencing is closely associated with histone modifications such as methyl-H3-K9. In this regard, the DNA demethylating agent 5-aza-2'-deoxycytidine (5-aza-CdR) and the HDAC inhibitor TSA reactivates expression of epigenetically silenced genes [26]. We examined the expression of these genes in cell lines after treatment with 5-aza-CdR, TSA, or both to test if the promoter hypermethylation-mediated down modulated gene expression can be reversed by demethylation and inhibition of HDACs. Of the five cell lines with *SLIT2 *promoter hypermethylation two (C-4I and SiHa) failed to induce reactivation after 5-aza-CdR or TSA treatments. Two other cell lines (SW756 and HT-3) showed minimal reactivation after treatment with one or the other drug. ME-180 is the only cell line that showed reactivation comparable to normal expression (Fig. 3B). None of the four cell lines (CaSki, HT-3, SW756, and SiHa) with *SLIT1 *methylated promoters showed reactivation (Fig. 3C). The *SLIT3 *gene failed to reactivate in two (CaSki and SiHa) of four methylated cell lines. The other two (SW756 and HeLa) cell lines showed only minimal reactivation after 5-aza-CdR treatment but not with TSA (Fig. 3C). The *ROBO1 *gene showed reactivated expression only in one of two (CaSki and ME-180) methylated cell lines (Fig. 3B). Thus, these data indicate that the demethylation of promoters of Slit-Robo pathway genes do not effectively reactivate gene expression. This failure or inappropriate reactivation of gene expression after 5-aza-CdR or HDAC treatments can be due to number of experimental problems such as aged buffered 5-aza-CdR or inadequate periods and concentrations of drug exposure. We ruled out these possibilities by using fresh 5-aza-CdR, varying drug concentrations (5 μg and 10 μg) and period of exposure (5 to 10 days), and in triplicate assays. The effect of drug treatment on demethylation was also confirmed by MSP in which the amplification of methylated allele was either completely absent or highly decreased and a reappearance of unmethylated alleles in a biallelically methylated cell lines (Fig. 5). Our cloning and sequencing analysis of 5-aza-CdR treated and bisulphate-converted DNAs also showed a rate of 33–65% demethylated CpG sites of *SLIT2* gene (data not shown).

Although the role of demethylating drugs that target transcriptional repressor complexes in tumors remains poorly understood, it is known that the interaction of receptors and their cognate ligands is critical in mediating gene activation[27]. The present observation of inefficient reactivation of Slit-Robo pathway genes after treatment with 5-aza-CdR in CC may be due to concomitant promoter hypermethylation of receptors and ligands resulting in failure of ligand-receptor interactions. Also, it has been shown that DNMT inhibitor 5-aza-CdR treatment has been shown to induce reactivation of only a limited number of genes in a tissue and pathway specific manner [28]. Based on this, Karpf et al. proposed that the mechanism of transcriptional regulation of 5-aza-CdR-mediated gene reactivation requires both a reversal of hypermethylation and the presence of trans-factors that mediate the activation of hypomethylated target promoters. In the present study, we show that the reversal of promoter hypermethylation of Slit-Robo pathway genes could be achieved after 5-aza-CdR treatment. However, we were unable to simultaneously achieve the gene re-activation. These data, thus, suggest that the promoter methylation-mediated activation of Slit-Robo pathway also requires critical upstream transcriptional regulators. The identification of such promoter specific transcriptional activators of Slit-Robo genes is essential to understand the role of hypemethylation of this pathway and to fully realize the scope of 5-aza-CdR-mediated gene activation. Whether such a phenomenon of Slit-Robo pathway regulation is restricted to CC or exists in other tumor types remains unknown.

## Conclusion

The present study identified a high frequency of promoter hypermethylation of Slit-Robo pathway genes in invasive CC and the associated precancerous lesions. These data, thus, suggest that Slit-Robo pathway inactivation significantly contribute to the pathogenesis of CC. These results provide new insights into possible pathogenic mechanisms in CC transformation and may have clinical implications in designing epigenetic-based therapy in the treatment of advanced stage CC. The occurrence of promoter hypermethylation in precancerous lesions and their association with progression to invasive CC suggests that these alterations may serve as biomarkers of risk prediction in progression.

## Methods

### Patients, tumor tissues, and cell lines

A total of 119 samples of DNA derived from 110 at-diagnosis tumor biopsies from invasive CC and nine cell lines were used. The tumor biopsies were ascertained from patients evaluated at the Instituto Nacional de Cancerologia (Santa Fe de Bogota, Colombia), Department of Obstetrics and Gynecology of Friedrich Schiller University (Jena, Germany), and Columbia University Medical Center (New York) after appropriate informed consent and approval of protocols by institutional review boards. The primary tumors were clinically classified as FIGO stage IB (27 tumors), IIB (31 tumors), IIIB (47 tumors), and IV (5 tumors). Histologically, 105 tumors (Age range 27–85 yrs; mean 49 yrs) were classified as squamous cell carcinoma (SCC) and five as adenocarcinoma (AC). Clinical information was collected from most patients as described [29]. Cervical swabs from 151 cases were collected in phosphate buffered saline from patients attending the Gynecologic Oncology Clinic at Columbia University Medical Center, New York, after appropriate informed consent. Forty-one of these were diagnosed cytologically as normal (Age range 16–74 yrs; mean 35.4 yrs) with no previous history of SIL, 62 as low-grade SIL (Age range 14–66 yrs; mean 29.7 yrs) and 48 as high-grade SIL (Age range 19–75 yrs; mean 39.2 yrs). In addition, we utilized 10 normal (Age range 41–64 yrs; mean 51.1 yrs) cervical epithelial cell preparations derived from hysterectomy specimens as normal controls. The CC cell lines HeLa, SiHa, SW756, C-4I, CaSki, C-33A, HT-3, MS751 and ME-180 were obtained from the American Type Culture Collection (Manassas, VA), and were grown according to the supplier's recommendations. DNA and/or RNA were isolated from frozen tumor tissues or cultured cells by standard methods. RNA was obtained from 10-micron sections with H&E staining of adjacent sections to evaluate tumor content. Specimens that contained more than 70% tumor cells were used for RNA preparation.

### Loss of Heterozygosity (LOH) analysis and HPV detection

LOH analysis was performed using STS primers for D4S1593, D4S1562, D4S2946, D4S1525, D3S1542, D3S3681, D3S3031, and D3S3508 obtained from Invitrogen (Carlsbad, CA) using standard methods [13,30]. Human papillomavirus types were identified as described earlier [29].

### Methylation Specific PCR (MSP) and sequencing

Genomic DNA was treated with sodium bisulphite as described [29]. Placental DNA treated in vitro with *Sss*I methyltransferase (New England BioLabs, Beverly, MA) and normal lymphocyte DNA converted with sodium bisulphite was used as methylated and unmethylated controls, respectively. Primers used for amplification of methylated and unmethylated promoters for each of the genes are shown in Table 1. PCR products were run on 2% agarose gels and visualized after ethidium bromide staining. All MSP experiments were performed three times and the promoter hypermethylation was considered positive only when confirmed twice. MSP products were either directly sequenced or sub-cloned into pCR2.1-TOPO (Invitrogen) followed by sequencing multiple clones using primers common to both methylated and unmethylated templates (Table 1).

### Drug treatment

Cells in culture were treated with 5 or 10 μM of 5-Aza-2'deoxycytidine (5-aza-CdR) for 5 to 10 days and 100–500 nM of Trichostatin A (TSA) for 24 hours as described [29].

### RT-PCR analysis

Total RNA isolated from treated and untreated cell lines, tumor tissues, and eight normal cervix uteri (three obtained from different commercial sources and five from hysterectomy specimens) was reverse transcribed as described [29]. A multiplex semi-quantitative analysis of gene expression was performed in replicate in three independent experiments as described [29]. A given gene was considered down regulated in a tumor when the level of mRNA was less than two standard deviations, except for *ROBO1 *in untreated cells, of the values obtained from the normal cervix. Primers used in the present study are shown in Table 1.

### Statistical analysis

Statistical analysis was performed using a Chi-square test.

## Competing interests

The author(s) declare that they have no competing interests.

## Authors' contributions

GN carried out all molecular genetic studies. CG participated in the MSP, cloning and sequencing. HA-P, AMK, AS, MD, MM, BP participated in collection of tissues, clinical information, and critical reading of the manuscript. VVM conceived the study, participated in its design and coordination and draft of the manuscript. All authors read and approved the final manuscript.
